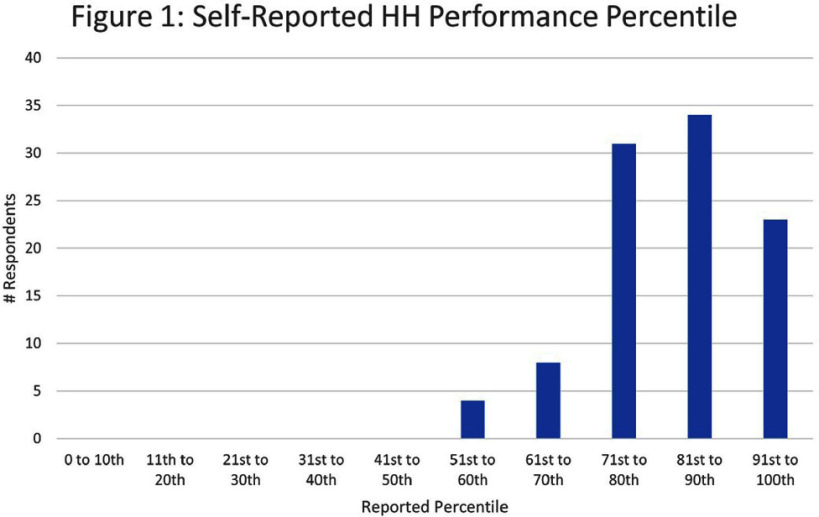# Overestimation of Personal Hand Hygiene Performance and Barriers to Hand Hygiene among Healthcare Providers

**DOI:** 10.1017/ash.2025.336

**Published:** 2025-09-24

**Authors:** Sonia Srikanth, Patrick Ching, Barry Rittmann, Laura Pedersen, Haley Brown, Tina Olkonen, Ryan Wooten, Kaila Cooper, Michelle Doll

**Affiliations:** 1 VCU School of Medicine; 2 Virginia Commonwealth University; 3 VCU Health; 4 Nursing VCU Health

## Abstract

**Introduction:** Hand hygiene (HH) performance in our facility declined during the COVID-19 pandemic and failed to return to baseline despite a widespread education campaign and increased HH rounding. To better understand provider perceptions and inform future interventions, we conducted a survey examining self-perception of HH performance, factors determining HH practices, barriers to adherence, and burnout. **Methods:** The survey assessed self-perceived HH performance relative to peers, perceived opportunities for improvement, barriers to HH, factors causing variation in personal HH practice (workload/acuity, peers, time of day, patient characteristics), and self-reported burnout using a validated, single-item burnout scale. Surveys were conducted in-person with clinical providers on one high-performing and three lower-performing intensive care units. All available clinical team members were included; non-clinical staff were excluded. Self-perception of performance was compared by unit, role, years of experience, and burnout rating using the Kruskal-Wallis test. Analyses were completed in SAS 9.4, Cary, NC. **Results:** One hundred surveys were completed. One person declined. The majority of those surveyed believed themselves to be in the top quartile of HH performers (87%). The actual HH compliance measured on these 4 units for 1/2024-11/2024 was 65% based on 7726 total directly observed opportunities. No one selected bottom or bottom 3rd quartiles. Figure 1 shows responses by percentile self-ranking. There was no difference in perceived performance by unit (p=0.4006), years of experience (p=0.9679), or burnout (p=0.2621). Non-clinical “other” type providers perceived performance to be slightly higher than clinical provider types: mean 91st percentile versus 82nd for prescribing providers, 84th for students, and 81st for nurses/nurse assistants, p=0.0353. Empty HH dispensers was the most frequent barrier cited, by 77%. A point prevalence survey on these 4 units completed the week after the survey ended verified that 22% (25/113) of dispensers were empty, however, in all of the 25 except for 1, there was a filled dispenser within 8 feet of the empty one. **Discussion:** HH performance was perceived to be better than average by the majority of inpatient healthcare providers (HCP) surveyed, despite data from these units indicating opportunities. Empty dispensers were consistently cited as a barrier, but likely could have been surmounted by a few steps based on the locations of the next available filled dispensers. Further improvement in HH will be difficult without efforts to move perception closer to reality for individual HCPs. Video footage or re-enactments of an observed care episode may help identify opportunities for improvement.

**Figure:**